# Complicating autoimmune diseases in myasthenia gravis: a review

**DOI:** 10.3109/08916934.2015.1030614

**Published:** 2015-04-27

**Authors:** Aliona Nacu, Jintana Bunpan Andersen, Vitalie Lisnic, Jone Furlund Owe, Nils Erik Gilhus

**Affiliations:** ^a^ Department of Neurology, Haukeland University HospitalBergenNorway; ^b^ Department of Neurology, State University of Medicine and Pharmacy “Nicolae Testemitanu”ChişinauRepublic of Moldova; ^c^ Department of Clinical Medicine, University of BergenBergenNorway

**Keywords:** Autoimmune disease, comorbidity, myasthenia gravis

## Abstract

Myasthenia gravis (MG) is a rare autoimmune disease of skeletal muscle endplates. MG subgroup is relevant for comorbidity, but usually not accounted for. MG patients have an increased risk for complicating autoimmune diseases, most commonly autoimmune thyroid disease, systemic lupus erythematosus and rheumatoid arthritis. In this review, we present concomitant autoimmune disorders associated with the different MG subgroups, and show how this influences treatment and prognosis. Concomitant MG should always be considered in patients with an autoimmune disorder and developing new neuromuscular weakness, fatigue or respiratory failure. When a second autoimmune disorder is suspected, MG should be included as a differential diagnosis.

## Introduction

Myasthenia gravis (MG) is a rare autoimmune disease caused by autoantibodies against neuromuscular junction proteins; the nicotinic acetylcholine receptor (AChR), the muscle specific tyrosine kinase (MuSK) or the low-density lipoprotein receptor-related protein 4 (LRP4) [1,2]. MG prevalence of 100–200 per million is similar in most populations [2]. The onset of MG is influenced by age and gender in a bimodal fashion. Females are more commonly affected below the age of 50 years, while males are slightly more frequently affected in late onset MG [3,4]. With improved diagnosis and prolonged survival, the prevalence is increasing, especially in the elderly [5–7], also previous under-diagnosed in the elderly. Patients with autoimmune MG can be classified into seven subgroups, each with distinctive autoantibodies and clinical features (Table 1). MG subgroup is highly relevant for comorbidity, but usually not accounted for. MG treatment may influence the risk for other disorders. The course of MG is often complicated by concomitant disease.

**Table 1.** Classification of MG subgroups and common associated autoimmune disorders.

MG subgroup		Age of onset (years)	Muscle auto-antibodies	Thymus involvement	Affected muscles	Associated autoimmune disorder
I	Ocular	All	AChR (50%)	Rarely	Ocular	ATD
II	Early onset	<50	AChR (100%)	Hyperplasia	Generalized	ATD, SLE, type 1 DM, AAT, GCM, NMO, myositis, PRCA, autoimmune hepatitis, Sjögren’s syndrome, Addison’s disease, dermatomyositis/polymyositis, GBS
III	Late onset	>50	AChR (100%), titin (60%), RyR (14%)	Atrophy	Generalized	Hashimoto’s disease, SLE, MS
IV	Thymoma	All	AChR (100%), titin (90%), RyR (75%)	Lympho-epithelioma	Ocular, bulbar, neck, generalized	SLE, neuromyotonia, Sjögren’s syndrome, autoimmune haemolytic anemia, POEMS syndrome
V	MuSK	All	MuSK (100%)	No	Ocular, bulbar, facial, generalized	SLE, pemphigus foliaceus, pemphigus vulgaris, MS
VI	LRP4	All	LRP4 (100%)	No	Bulbar	LEMS, NMO
VII	Antibody negative	All	Low affinity ab, low ab concentration, other junction protein-ab, non-ab, heterogenous	Variable	Ocular, generalized	

MG, myasthenia gravis; AchR, acetylcholine receptor; RyR, ryanodine receptor; MuSK, muscle specific tyrosine kinase; LRP4, low density lipoprotein receptor-related protein 4; ab, antibody; ATD, autoimmune thyroid disease; SLE, systemic lupus erythematosus, NMO, neuromyelitisoptica; DM, diabetes mellitus; AAT, alopecia areata totalis; GCM, giant cell myocarditis; PRCA, pure red cell aplasia; GBS, Guillain–Barre syndrome, MS, multiple sclerosis; POEMS, polyneuropathy, organomegaly, endocrinopathy, monoclonal plasma-proliferative disorder, skin changes; LEMS, Lambert Eaton myasthenic syndrome.

Approximately 5% of the population is affected by one or more autoimmune disorders, and the prevalence is higher in females than in males. Patients affected by one autoimmune disorder have a higher risk of developing a second one. MG patients have an increased risk of other autoimmune disorders compared to the non-MG population [8,9]. The frequency of a second autoimmune disorder is 13–22% in MG patients, highest for females and early onset MG (EOMG) [8,9]. Among 100.000 patients with autoimmune disorders, only 35 have MG [10]. However, 0.2% of patients with thyroid disease have MG, much higher than expected by chance [11], whereas 0.1% of patients with multiple sclerosis (MS) have MG [12]. In a systematic review, autoimmune thyroid disease (ATD) was the most frequent of 23 associated autoimmune disorders, occurring in 10% of MG patients [13]. Other common autoimmune associates with MG are systemic lupus erythematosus (SLE) (1-8%), rheumatoid arthritis (RA) (4%), dermato-/polymyositis and Addison’s disease [8,13–16].

Genetic studies on the development of autoimmune disorders reveal common as well as distinctive susceptibility genes, most strongly at the human leukocyte antigen (HLA) locus [17]. Genome-wide association studies (GWAS) on EOMG shed light on specific additional genetic hot spots for MG [18]. However, the exact pathogenesis, genetic mechanisms and trigger factors underlying MG as well as MG in combination with other autoimmune disorders are still unknown. Patient selection, follow-up time and sensitivity in finding concomitant disease vary in studies of comorbidity. Correct characterization of autoimmune comorbidity will improve insight in MG pathogenesis and optimize the combined treatment of the disorders.

## Associated autoimmune disorders in non-thymoma AChR-MG

AChR-MG represents 80% of MG patients, and AChR antibodies are found in both ocular and generalized MG [2]. Thyroid disease with thyroid-related antibodies and primary Sjögren’s syndrome related antibodies are seen in both ocular and generalized MG subgroups [19], most frequently in the ocular group [20]. In 8% of patients with thyroid-associated ophtalmopathy, AChR antibodies are found [21], possibly due to common autoimmune targets in the eye muscle and/or genetic factors [22,23]. Thymic thyrotropin receptor may act as an auto-antigen in Graves’ disease when associated with thymic hyperplasia [24,25]. MG in association with ATD has a younger age of onset, mild clinical expression, rare MG crisis, lower levels of AChR antibodies and lower frequency of thymic changes [20,26,27].

EOMG with AChR antibodies is often clinically mild, has a higher frequency of thymic hyperplasia and lower positivity for AChR antibodies compared with late onset MG (LOMG) [20,27,28]. In EOMG, ATD, SLE, type 1 diabetes mellitus (DM), neuromyelitis optica, alopecia areata totalis, giant cell myocarditis, myositis, pure red cell aplasia, autoimmune hepatitis all appear more frequently than expected [16,19,29–34], especially in females [35,36]. Recently, a large Swedish population-based study with over 2000 MG patients identified dermato-/polymyositis, SLE, Addison’s disease, Guillain–Barre syndrome and juvenile RA as the most frequent associated autoimmune disorders in MG, especially in the EOMG subgroup [8]. Furthermore, ATD and DM (both type 1 and 2) occur more frequently in patients with AChR antibodies than in those without such antibodies [30]. Selective immunoglobulin A deficiency, the most common primary immunodeficiency in Caucasians, is associated with other autoimmune disorders including MG [29]. EOMG is strongly associated with the haplotype HLA-B8-DR3 [30,37], which is also associated with ATD [38], type 1 DM [39], Sjögren’s syndrome, inclusion body myositis [40], dermato-/polymyositis, SLE and Addison’s disease [8].

LOMG has a lower frequency of autoimmune overlap than the other MG subgroups [41]. These are the data from a retrospective study of 112 MG patients from 1971 to 2006 conducted in a single neurological department. Nevertheless, both SLE and ATD are more frequent in the LOMG subgroup than in control populations, especially Hashimoto’s disease. Their clinical MG features are similar to those of MG patients without autoimmune disorders [28]. Most other autoimmune disorders also occur with a higher frequency in LOMG than in the non-MG population [42]. The prevalence of antiphospholipid antibodies is increased in LOMG, and this should influence decisions regarding thrombosis prophylaxis, for example during MG exacerbations [14].

Myopathy is more common in LOMG than in EOMG, and is associated with anti-titin antibodies [43]. The increased weakness seen in some elderly MG patients can be caused by an immunologically mediated myopathy, leading to a poorer prognosis. In MG patients with inflammatory myopathies, the disease can be severe with bulbar symptoms, MG crisis and often invasive thymoma [44]. There is evidence of CD8+ lymphocyte infiltration of skeletal muscle and presence of anti-striational antibodies [44].

## Associated autoimmune disorders in thymoma-MG

The thymus plays an important role for several MG subgroups, and 15% of all MG patients have a thymoma. Patients with MG and thymoma always have anti-AChR antibodies [7]. This reflects that the neoplastic thymus transformation initiates the autoimmune response to AChR [45]. In contrast, the presence of MuSK antibodies in the thymoma-MG subgroup is extremely rare, and it is presumed that thymus is not involved in the pathogenesis of MuSK-MG [46].

Thymomas have a high frequency of autoimmune associated disorders (45%), infections (24%) and both in combination (10%). More than one autoimmune disorders are found in 20% of the cases [47]. However, thymomas are related to only a few autoimmune disorders [48–50], the most frequent by far being MG, occurring in 30% of all thymoma cases [47]. In a series of nearly 600 patients with thymoma, 71% had an associated condition, 44% of these had MG, 21% cytopenias, 17% cancer, 6% hypogammaglobulinemia, 5% polymyositis and 2% SLE [51]. In contrast to other MG subgroups, ATD is uncommon in thymoma-MG [52]. Thymoma-MG can be associated also with neuromyotonia, Sjögren’s syndrome and autoimmune hemolytic anemia [53].

Thymomas are present at debut of MG. MG can develop many years after the detection of a thymoma. Thymomas can be very large at MG debut, reflecting their presence for many years. MG patients with thymoma are even susceptible to develop autoimmune disorders after thymectomy with removal of the tumor [33,54]. Thymomas seem to have antigen-specific, but also antigen-unspecific, correlation to autoimmunity. T cells of the TCRαβ lineage occur more frequently in thymomas associated with MG [24,55]. Thymomas produce autoantigen specific T cells by abnormal positive or negative T cell selection, leading to MG, as well as some other autoimmune disorders. These T cells then leave the thymoma to induce autoimmune disease [56]. Risk of thymoma in MG is associated with the TT homozygous genotype and the DNMT3B-579T allele [56]. This genotype does not occur with increased frequency in MG patients without thymoma, even after stratification into MG subgroups [57].

Autoimmune heart disease, especially myocarditis and giant cell myocarditis, has a higher incidence in the thymoma-MG subgroup, confirmed pathologically with lymphocyte infiltration and giant cells in the heart [58]. Heart muscle antibodies are related only to thymoma-MG and LOMG subgroups [59]. Antibodies such as those against voltage-gated potassium channel (VGKC) Kv1.4, titin and ryanodine receptor have been shown to react with heart muscle in MG patients [58]. A possible association between anti-Kv1.4 antibodies and cardiac involvement, especially myocarditis, was recently described [60], the disease characterized by heart failure and arrhythmias together with limb and paraspinal muscle weakness. Giant cell myocarditis is potentially fatal and should be paid special attention [61], with immediate admission to an intensive care unit and start of immunosuppressive treatment [62].

Neoplasia, hyperplasia and age-related degeneration of the thymus are relevant when considering MG treatment. In some patients, immunosuppressive MG treatment, including thymectomy, may disturb the balance of immunity and lead to additional disorders [11,54,63].

The T cell repertoire is altered after thymectomy [33]. Theoretically this could both increase and decrease the risk for a second autoimmune disorder. The MG response to thymectomy is the same with and without a second autoimmune disease. However, thymectomy has no influence on the second autoimmune disease [11,64]. Studies suggesting that thymectomy may increase the susceptibility for an additional autoimmune disorder, and especially for SLE [65,66], are based on a small number of cases and with a selection bias. MG appears in about 1% of the patients post thymomectomy, and surgery may not prevent postoperative MG [67].

## Associated autoimmune disorders in MuSK-MG

MuSK antibodies are present in 10–70% of all MG patients without AChR antibodies [2,68,69], and is clinically more severe than other MG subtypes with involvement of facial, bulbar and upper body muscles, sometimes with muscle atrophy [2,69,70]. MuSK-MG is more prevalent in Mediterranean countries and less in Northern Europe [71].

Very few studies have reported the frequency of associated autoimmune disorders in MuSK-MG specifically. Association with HLA-DR14-DQ5 occurs for MuSK-MG, similar to pemphigus foliaceus and pemphigus vulgaris [72–74]. Interleukins 4 and 13, secreted by Th2-type T cells, are involved in MuSK-MG and pemphigus [75]. MuSK-MG and SLE can be found in the same patient [19]. An association between relapsing–remitting MS and MuSK-MG has been suggested [76]. Rituximab represents an effective treatment [77].

Coexistence of AChR and MuSK antibodies has been reported in only five patients [78–82]. In one case, AChR, MuSK and VGKC antibodies co-occurred with MG and Morvan's syndrome without any thymoma [78]. In the other four cases, the patients were AChR antibody positive at disease onset, but became MuSK antibody positive after thymectomy. D-penicillamine can induce both AChR and MuSK antibody-positive MG, a rare phenomenon which is reversible after discontinuation of the treatment [83].

## Associated autoimmune disorders in LRP4-MG

LRP4 is a receptor for agrin and important in the formation of the neuromuscular junction [1]. LRP4 autoantibodies are reported in up to 50% of MG patients without AChR or MuSK antibodies, and even in a few patients with MuSK-MG [1,84,85]. Patients with exclusively bulbar symptoms may have double seropositive MG (LRP4 and AChR) [86]. Case reports of concomitant autoimmune disease in the LRP4-MG subgroup are still lacking. However, LRP4 antibodies have been found in Lambert Eaton myasthenic syndrome and neuromyelitis optica [68]. Thymoma has not been observed in LRP4-MG [85]. As for MuSK-MG, no role of the thymus has been established in the LRP4-MG pathogenesis [46,87]. The IgG1 subclass, a complement activator, predominates for the LRP4 antibodies. In AChR-MG, the IgG1 and IgG3 subclasses predominate. In contrast, MuSK-MG predominantly has IgG4 which does not activate complement [1,68]. The clinical presentation of LRP4-MG is milder compared with other MG subgroups, and the response to therapy is good. A significant number of double positives (7.5% AChR/LRP4-MG and 15% MuSK/LRP4-MG) were recently described, this being more severe cases [88].

## Autoimmune overlap and common etiologic factors

Autoimmunity arises from an inappropriate response against tissue elements normally present, leading to complex chronic diseases characterized by the loss of immune tolerance to self-antigens [89]. Autoimmune overlap reflects common pathogenic mechanisms [10,90]; immunological factors leading to activation of autoreactive B and T cells, genetic susceptibility involving specific candidate genes and epigenetic factors [91]. The genetic interplay is highly recognized in the development of autoimmune disorders, due to high concordance rates reported from twin studies and observations of candidate genes, particularly HLA genes, strongly associated with autoimmune diseases [91]. Understanding the common mechanisms in the pathophysiology of MG and other autoimmune disorders is important for an effective and target-directed treatment [92]. Associated genetic variants in autoimmune disease are shared between systemic and organ-specific autoimmune diseases [93]. Disease models for autoimmune diseases such as MG, RA, MS, DM, autoimmune myocarditis, ATD and uveitis have been developed using HLA alleles transgenic mice [94]. This humanized transgenic mice models help in understanding the role of HLA genes in the pathogenesis of MG and other autoimmune diseases.

The genetic influence of the major histocompatibility complex (MHC) in MG is thought to be mediated through a single signal in the class I region, induced in the vicinity of the HLA complex protein 5 gene located between MICA and MICB [95,96]. EOMG and other autoimmune disorders are associated with the HLA A1-B8-DR3-DQ2 haplotype. GWAS analysis using 649 EOMG cases and 2596 controls showed a strong association to the HLA-B * 08 locus allele and to two additional loci outside MHC: the PTPN22 gene and TNFAIP3-interacting protein 1 (TNIP1) [18]. The HLA-B * 08 represents a risk allele for most autoimmune diseases [97]. *In vivo* and *in vitro* studies suggest that TNIP1 plays a crucial role in regulating several cellular pathways involved in immune-related disorders [92]. TNIP1 is a risk allele for RA, psoriasis, Sjogren's syndrome and SLE [98–100].

A recent study highlights the contribution of the HLA-B8DR3 haplotype in MG, especially in female EOMG [8]. The B8DR3 haplotype also increases the risk for SLE, Addison's disease and dermatomyositis/polymyositis in MG [8]. An association between the MS-associated allele DRB1*1501 and the LOMG subgroup was reported, illustrating how different MG subtypes belong to different genetic clusters [8]. An association between HLA-C*0701, DRB1*15:01, DRB1*16, DQB1*05:02 and LOMG has been found [101,102].

It will be of interest to determine whether endophenotypes, such as the identity of the autoantibody (AChR vs. MuSK vs. titin), the presence of thymoma or the occurrence of associated autoimmune diseases are associated with distinctive HLA-region signals. MHC class II gene associations have been reported specifically for patients with anti-MuSK or anti-titin antibodies [103]. The HLA class II allele HLA-DRB1*16,-DRB1*14 and -DQB1*05 allele is linked to MuSK-MG [73,103,104], and also HLA-DRB1*03 appears to have a distinguishing role for this subgroup compared to AChR-MG [103]. Genetic susceptibility and MG subgroups vary between populations. More than 90% of Southern Han Chinese ocular MG patients had the DQ9 haplotype [105].

Some non-major MHC genes identified as important susceptibility genes in MG are shared with other autoimmune disorders. The protein tyrosine phosphatase non-receptor 22 (PTPN22) R620W gene polymorphism is a general risk factor for autoimmune diseases with an increased production of auto-antibodies [18] and predisposes MG and EOMG in particular for additional autoimmune diseases [29,106]. PTPN22 showed the strongest association to the thymoma-MG and EOMG subgroups [107].

The lack of association of PTPN22 R620W polymorphism with MuSK-MG and antibody negative-MG subgroups [108] emphasizes the different genetic background for MG subgroups. Young EOMG (debut < 20 years) is strongly associated with the nicotinic cholinergic receptor alpha 1 locus which encodes the α-subunit of the AChR and interacts with the autoimmune regulator [109]. *CTLA4* gene polymorphisms are associated with MG and also other autoimmune diseases such as type 1 DM, ATD, SLE, RA and celiac disease [92,110–115]. The cathepsin L2 is associated with the EOMG subgroup and type 1 DM [36].

## Conclusions

Autoimmune overlap between MG and other autoimmune disorders reflects common pathogenic mechanisms. ATD is the most frequent comorbidity. Comorbidity is linked to MG subgroup. It is a paradox that LOMG and thymoma-MG have a broad antimuscle antibody response, but the immune response usually being confined to muscle, whereas EOMG has a restricted antimuscle response against AChR only, but often has a broad general response against several organ tissues. MG in association with other autoimmune disorders has a less favorable prognosis. It is important to consider coexisting MG in patients with autoimmune disorders and neuromuscular weakness, fatigue and respiratory failure.

## References

[CIT0001] Zhang B., Tzartos J. S, Belimezi M (2012). Autoantibodies to lipoprotein-related protein 4 in patients with double-seronegative myasthenia gravis. Arch. Neurol..

[CIT0002] Gilhus N. E. (2009). Autoimmune myasthenia gravis. Expert Rev. Neurother..

[CIT0003] Keesey J. C. (2004). Clinical evaluation and management of myasthenia gravis. Muscle Nerve..

[CIT0004] Grob D., Brunner N, Namba T, Pagala M (2008). Lifetime course of myasthenia gravis. Muscle Nerve..

[CIT0005] Jacob S., Viegas S, Lashley D, Hilton-Jones D (2009). Myasthenia gravis and other neuromuscular junction disorders. Pract. Neurol..

[CIT0006] Alkhawajah N. M., Oger J (2013). Late-onset myasthenia gravis: a review when incidence in older adults keeps increasing. Muscle Nerve..

[CIT0007] Aarli J. A (2008). Myasthenia gravis in the elderly: is it different?. Ann. N. Y. Acad. Sci..

[CIT0008] Fang F., Sveinsson O, Thormar G (2014). The autoimmune spectrum of myasthenia gravis: a Swedish population-based study. J. Intern. Med..

[CIT0009] Christensen P. B., Jensen T. S, Tsiropoulos I (1995). Associated autoimmune diseases in myasthenia gravis. A population-based study. Acta Neurol. Scand..

[CIT0010] Sardu C., Cocco E, Mereu A (2012). Population based study of 12 autoimmune diseases in Sardinia, Italy: prevalence and comorbidity. PLoS One.

[CIT0011] Tellez-Zenteno J. F., Cardenas G, Estanol B (2004). Associated conditions in myasthenia gravis: response to thymectomy. Eur. J. Neurol..

[CIT0012] Ramagopalan S. V., Anderson C, Sadovnick A. D, Ebers G. C (2007). Genomewide study of multiple sclerosis. N. Engl. J. Med..

[CIT0013] Mao Z. F., Yang L. X, Mo X. A (2011). Frequency of autoimmune diseases in myasthenia gravis: a systematic review. Int. J. Neurosci..

[CIT0014] Jallouli M., Saadoun D, Eymard B (2012). The association of systemic lupus erythematosus and myasthenia gravis: a series of 17 cases, with a special focus on hydroxychloroquine use and a review of the literature. J. Neurol..

[CIT0015] Thorlacius S., Aarli J. A, Riise T (1989). Associated disorders in myasthenia gravis: autoimmune diseases and their relation to thymectomy. Acta Neurol. Scand..

[CIT0016] Sthoeger Z., Neiman A, Elbirt D (2006). High prevalence of systemic lupus erythematosus in 78 myasthenia gravis patients: a clinical and serologic study. Am. J. Med. Sci..

[CIT0017] Gilhus N. E., Nacu A, Andersen J. B, Owe J. F (2015). Myasthenia gravis and risks for comorbidity. Eur. J. Neurol..

[CIT0018] Gregersen P. K., Kosoy R, Lee A. T (2012). Risk for myasthenia gravis maps to a (151) Pro→Ala change in TNIP1 and to human leukocyte antigen-B*08. Ann. Neurol..

[CIT0019] Nakamura H., Usa T, Motomura M (2008). Prevalence of interrelated autoantibodies in thyroid diseases and autoimmune disorders. J. Endocrinol. Invest..

[CIT0020] Chen Y. L., Yeh J. H, Chiu H. C (2013). Clinical features of myasthenia gravis patients with autoimmune thyroid disease in Taiwan. Acta Neurol. Scand..

[CIT0021] Jacobson D. M (1995). Acetylcholine receptor antibodies in patients with Graves' ophthalmopathy. J. Neuroophthalmol..

[CIT0022] Marino M., Barbesino G, Pinchera A (2000). Increased frequency of euthyroid ophthalmopathy in patients with Graves' disease associated with myasthenia gravis. Thyroid..

[CIT0023] Kaminski H. J., Li Z, Richmonds C (2003). Susceptibility of ocular tissues to autoimmune diseases. Ann. N. Y. Acad. Sci..

[CIT0024] Yamanaka K., Nakayama H, Watanabe K, Kameda Y (2006). Anterior mediastinal mass in a patient with Graves' disease. Ann. Thorac. Surg..

[CIT0025] Stefan M., Wei C, Lombardi A (2014). Genetic-epigenetic dysregulation of thymic TSH receptor gene expression triggers thyroid autoimmunity. Proc. Natl. Acad. Sci USA..

[CIT0026] Garlepp M. J., Dawkins R. L, Christiansen F. T (1981). Autoimmunity in ocular and generalised myasthenia gravis. J. Neuroimmunol..

[CIT0027] Mistry N., Wass J, Turner M. R (2009). When to consider thyroid dysfunction in the neurology clinic. Pract. Neurol..

[CIT0028] Kanazawa M., Shimohata T, Tanaka K, Nishizawa M (2007). Clinical features of patients with myasthenia gravis associated with autoimmune diseases. Eur. J. Neurol..

[CIT0029] Ramanujam R., Piehl F, Pirskanen R (2011). Concomitant autoimmunity in myasthenia gravis – lack of association with IgA deficiency. J. Neuroimmunol..

[CIT0030] Toth C., McDonald D, Oger J, Brownell K (2006). Acetylcholine receptor antibodies in myasthenia gravis are associated with greater risk of diabetes and thyroid disease. Acta Neurol. Scand..

[CIT0031] Leite M. I., Coutinho E, Lana-Peixoto M (2012). Myasthenia gravis and neuromyelitis optica spectrum disorder: a multicenter study of 16 patients. Neurology..

[CIT0032] Lakhal K., Blel Y, Fysekidis M (2008). Concurrent Graves disease thyrotoxicosis and myasthenia gravis: the treatment of the former may dangerously reveal the latter. Anaesthesia..

[CIT0033] Suzuki S., Shimoda M, Kawamura M (2005). Myasthenia gravis accompanied by alopecia areata: clinical and immunogenetic aspects. Eur. J. Neurol..

[CIT0034] Iyer A., Elsone L, Appleton R, Jacob A (2014). A review of the current literature and a guide to the early diagnosis of autoimmune disorders associated with neuromyelitis optica. Autoimmunity.

[CIT0035] Poulas K., Tsibri E, Kokla A (2001). Epidemiology of seropositive myasthenia gravis in Greece. J. Neurol. Neurosurg. Psychiatry.

[CIT0036] Viken M. K., Sollid H. D, Joner G (2007). Polymorphisms in the cathepsin L2 (CTSL2) gene show association with type 1 diabetes and early-onset myasthenia gravis. Hum. Immunol..

[CIT0037] Giraud M., Beaurain G, Yamamoto A. M (2001). Linkage of HLA to myasthenia gravis and genetic heterogeneity depending on anti-titin antibodies. Neurology.

[CIT0038] Kraemer M. H., Donadi E. A, Tambascia M. A (1998). Relationship between HLA antigens and infectious agents in contributing towards the development of Graves' disease. Immunol. Invest..

[CIT0039] Barker J. M., Barriga K. J, Yu L, Diabetes Autoimmunity Study in the Young (2004). Prediction of autoantibody positivity and progression to type 1 diabetes: Diabetes Autoimmunity Study in the Young (DAISY). J. Clin. Endocrinol. Metab..

[CIT0040] Badrising U. A., Schreuder G. M, Giphart M. J (2004). Associations with autoimmune disorders and HLA class I and II antigens in inclusion body myositis. Neurology.

[CIT0041] Matsui N., Nakane S, Nakagawa Y (2009). Increasing incidence of elderly onset patients with myasthenia gravis in a local area of Japan. J. Neurol. Neurosurg. Psychiatry.

[CIT0042] Klein R., Marx A, Strobel P (2013). Autoimmune associations and autoantibody screening show focused recognition in patient subgroups with generalized myasthenia gravis. Hum. Immunol..

[CIT0043] Somnier F. E., Skeie G. O, Aarli J. A, Trojaborg W (1999). EMG evidence of myopathy and the occurrence of titin autoantibodies in patients with myasthenia gravis. Eur. J. Neurol..

[CIT0044] Suzuki S., Utsugisawa K, Yoshikawa H (2009). Autoimmune targets of heart and skeletal muscles in myasthenia gravis. Arch. Neurol..

[CIT0045] Cavalcante P., Cufi P, Mantegazza R (2013). Etiology of myasthenia gravis: innate immunity signature in pathological thymus. Autoimmun. Rev..

[CIT0046] Marx A., Pfister F, Schalke B (2013). The different roles of the thymus in the pathogenesis of the various myasthenia gravis subtypes. Autoimmun. Rev..

[CIT0047] Holbro A., Jauch A, Lardinois D (2012). High prevalence of infections and autoimmunity in patients with thymoma. Hum. Immunol..

[CIT0048] Levy Y., Afek A, Sherer Y (1998). Malignant thymoma associated with autoimmune diseases: a retrospective study and review of the literature. Semin. Arthritis Rheum..

[CIT0049] Marx A., Muller-Hermelink H. K, Strobel P (2003). The role of thymomas in the development of myasthenia gravis. Ann. N. Y. Acad. Sci..

[CIT0050] Muller-Hermelink H. K., Marx A (2000). Thymoma. Curr. Opin. Oncol..

[CIT0051] Souadjian J. V., Enriquez P, Silverstein M. N, Pepin J. M (1974). The spectrum of diseases associated with thymoma. Coincidence or syndrome?. Arch. Intern. Med..

[CIT0052] Marino M., Ricciardi R, Pinchera A (1997). Mild clinical expression of myasthenia gravis associated with autoimmune thyroid diseases. J. Clin. Endocrinol. Metab..

[CIT0053] De Keyzer K., Peeters P, Verhelst C (2009). Autoimmune haemolytic anaemia associated with a thymoma: case report and review of the literature. Acta Clin. Belg..

[CIT0054] Suzuki S., Nogawa S, Tanaka K (2003). Initial predictors of development of pure red cell aplasia in myasthenia gravis after thymectomy. Clin. Neurol. Neurosurg..

[CIT0055] Inada K., Okumura M, Shiono H (2005). Role of positive selection of thymoma-associated T cells in the pathogenesis of myasthenia gravis. J. Surg. Res..

[CIT0056] Yapali S., Oruc N, Ilgun S (2012). Acute presentation of autoimmune hepatitis in a patient with myasthenia gravis, thymoma, Hashimoto thyroiditis and connective tissue disorder. Hepatol. Res..

[CIT0057] Coppede F., Ricciardi R, Denaro M (2013). Association of the DNMT3B-579G>T polymorphism with risk of thymomas in patients with myasthenia gravis. PLoS One.

[CIT0058] Suzuki S., Utsugisawa K, Nagane Y, Suzuki N (2011). Three types of striational antibodies in myasthenia gravis. Autoimmune Dis..

[CIT0059] Mygland A., Aarli J. A, Hofstad H, Gilhus N. E (1991). Heart muscle antibodies in myasthenia gravis. Autoimmunity.

[CIT0060] Suzuki S., Baba A, Kaida K (2014). Cardiac involvements in myasthenia gravis associated with anti-Kv1.4 antibodies. Eur. J. Neurol..

[CIT0061] Rosenstein E. D., Zucker M. J, Kramer N (2000). Giant cell myocarditis: most fatal of autoimmune diseases. Semin. Arthritis Rheum..

[CIT0062] Suzuki S., Utsugisawa K, Suzuki N (2013). Overlooked non-motor symptoms in myasthenia gravis. J. Neurol. Neurosurg. Psychiatry..

[CIT0063] Zonana M. F., Reyes E, Weisman A. K (2002). Coexistence of four autoimmune diseases in one patient: the kaleidoscope of autoimmunity. J. Clin. Rheumatol..

[CIT0064] Ratanakorn D., Vejjajiva A (2002). Long-term follow-up of myasthenia gravis patients with hyperthyroidism. Acta Neurol. Scand..

[CIT0065] Mevorach D., Perrot S, Buchanan N. M (1995). Appearance of systemic lupus erythematosus after thymectomy: four case reports and review of the literature. Lupus.

[CIT0066] Gerli R., Paganelli R, Cossarizza A (1999). Long-term immunologic effects of thymectomy in patients with myasthenia gravis. J Allergy Clin. Immunol..

[CIT0067] Kondo K., Monden Y (2005). Myasthenia gravis appearing after thymectomy for thymoma. Eur. J. Cardiothorac. Surg..

[CIT0068] Verschuuren J. J., Huijbers M. G, Plomp J. J (2013). Pathophysiology of myasthenia gravis with antibodies to the acetylcholine receptor, muscle-specific kinase and low-density lipoprotein receptor-related protein 4. Autoimmun. Rev..

[CIT0069] Evoli A., Tonali P. A, Padua L (2003). Clinical correlates with anti-MuSK antibodies in generalized seronegative myasthenia gravis. Brain..

[CIT0070] Verschuuren J. J., Palace J, Gilhus N. E (2010). Clinical aspects of myasthenia explained. Autoimmunity.

[CIT0071] Vincent A., Leite M. I, Farrugia M. E (2008). Myasthenia gravis seronegative for acetylcholine receptor antibodies. Ann. N. Y. Acad. Sci..

[CIT0072] McConville J., Farrugia M. E, Beeson D (2004). Detection and characterization of MuSK antibodies in seronegative myasthenia gravis. Ann. Neurol..

[CIT0073] Niks E. H., Kuks J. B, Roep B. O (2006). Strong association of MuSK antibody-positive myasthenia gravis and HLA-DR14-DQ5. Neurology..

[CIT0074] Niks E. H., Kuks J. B, Verschuuren J. J (2007). Epidemiology of myasthenia gravis with anti-muscle specific kinase antibodies in The Netherlands. J. Neurol. Neurosurg. Psychiatry..

[CIT0075] Tron F., Gilbert D, Mouquet H (2005). Genetic factors in pemphigus. J. Autoimmun..

[CIT0076] Sylvester J., Purdie G, Slee M (2013). Muscle-specific kinase antibody positive myaesthenia gravis and multiple sclerosis co-presentation: a case report and literature review. J. Neuroimmunol..

[CIT0077] Horvath B., Huizinga J, Pas H. H (2012). Low-dose rituximab is effective in pemphigus. Br. J. Dermatol..

[CIT0078] Diaz-Manera J., Rojas-Garcia R, Gallardo E (2007). Antibodies to AChR, MuSK and VGKC in a patient with myasthenia gravis and Morvan's syndrome. Nat. Clin. Pract. Neurol..

[CIT0079] Saulat B., Maertens P, Hamilton W. J, Bassam B. A (2007). Anti-musk antibody after thymectomy in a previously seropositive myasthenic child. Neurology.

[CIT0080] Sanders D. B., Juel V. C (2008). MuSK-antibody positive myasthenia gravis: questions from the clinic. J. Neuroimmunol.

[CIT0081] Kostera-Pruszczyk A., Kwiecinski H (2009). Juvenile seropositive myasthenia gravis with anti-MuSK antibody after thymectomy. J. Neurol..

[CIT0082] Rajakulendran S., Viegas S, Spillane J, Howard R. S (2012). Clinically biphasic myasthenia gravis with both AChR and MuSK antibodies. J. Neurol..

[CIT0083] Poulas K., Koutsouraki E, Kordas G (2012). Anti-MuSK- and anti-AChR-positive myasthenia gravis induced by d-penicillamine. J. Neuroimmunol..

[CIT0084] Pevzner A., Schoser B, Peters K (2012). Anti-LRP4 autoantibodies in AChR- and MuSK-antibody-negative myasthenia gravis. J. Neurol..

[CIT0085] Higuchi O., Hamuro J, Motomura M, Yamanashi Y (2011). Autoantibodies to low-density lipoprotein receptor-related protein 4 in myasthenia gravis. Ann. Neurol..

[CIT0086] Tsivgoulis G., Dervenoulas G, Tzartos S. J (2014). Double seropositive myasthenia gravis with acetylcholine receptor and lipoprotein receptor-related protein 4 antibodies. Muscle Nerve.

[CIT0087] Guptill J. T., Sanders D. B (2010). Update on muscle-specific tyrosine kinase antibody positive myasthenia gravis. Curr. Opin. Neurol..

[CIT0088] Zisimopoulou P., Evangelakou P, Tzartos J (2014). A comprehensive analysis of the epidemiology and clinical characteristics of anti-LRP4 in myasthenia gravis. J. Autoimmun..

[CIT0089] Davidson A., Diamond B (2001). Autoimmune diseases. N. Engl. J. Med..

[CIT0090] Waterfield M., Anderson M. S (2010). Clues to immune tolerance: the monogenic autoimmune syndromes. Ann. N. Y. Acad. Sci..

[CIT0091] Bach J. F. (2012). The etiology of autoimmune diseases: the case of myasthenia gravis. Ann. N. Y. Acad. Sci..

[CIT0092] Avidan N., Le Panse R, Berrih-Aknin S, Miller A (2014). Genetic basis of myasthenia gravis – a comprehensive review. J. Autoimmun..

[CIT0093] Zhernakova A., Withoff S, Wijmenga C (2013). Clinical implications of shared genetics and pathogenesis in autoimmune diseases. Nat. Rev. Endocrinol..

[CIT0094] Mangalam A. K., Rajagopalan G, Taneja V, David C. S (2008). HLA class II transgenic mice mimic human inflammatory diseases. Adv. Immunol..

[CIT0095] Liu X., Tian W, Li L, Cai J (2011). Characterization of the major histocompatibility complex class I chain-related gene B (MICB) polymorphism in a northern Chinese Han population: the identification of a new MICB allele, MICB*023. Hum. Immunol..

[CIT0096] Franciotta D., Cuccia M, Dondi E (2001). Polymorphic markers in MHC class II/III region: a study on Italian patients with myasthenia gravis. J. Neurol. Sci..

[CIT0097] Cho J. H., Gregersen P. K (2011). Genomics and the multifactorial nature of human autoimmune disease. N. Engl. J. Med..

[CIT0098] Adrianto I., Wang S, Wiley G. B (2012). Association of two independent functional risk haplotypes in TNIP1 with systemic lupus erythematosus. Arthritis Rheum..

[CIT0099] Lessard C. J., Li H, Adrianto I (2013). Variants at multiple loci implicated in both innate and adaptive immune responses are associated with Sjogren's syndrome. Nat. Genet..

[CIT0100] Ramirez V. P., Gurevich I, Aneskievich B. J (2012). Emerging roles for TNIP1 in regulating post-receptor signaling. Cytokine Growth Factor Rev..

[CIT0101] Maniaol A. H., Elsais A, Lorentzen A. R (2012). Late onset myasthenia gravis is associated with HLA DRB1*15:01 in the Norwegian population. PLoS One.

[CIT0102] Testi M., Terracciano C, Guagnano A (2012). Association of HLA-DQB1 *05:02 and DRB1 *16 alleles with late-onset, nonthymomatous, AChR-Ab-positive myasthenia gravis. Autoimmune Dis..

[CIT0103] Alahgholi-Hajibehzad M., Yilmaz V, Gulsen-Parman Y (2013). Association of HLA-DRB1 *14, -DRB1 *16 and -DQB1 *05 with MuSK-myasthenia gravis in patients from Turkey. Hum. Immunol..

[CIT0104] Bartoccioni E., Scuderi F, Augugliaro A (2009). HLA class II allele analysis in MuSK-positive myasthenia gravis suggests a role for DQ5. Neurology.

[CIT0105] Zhu W. H., Lu J. H, Lin J (2012). HLA-DQA1*03:02/DQB1*03:03:02 is strongly associated with susceptibility to childhood-onset ocular myasthenia gravis in Southern Han Chinese. J. Neuroimmunol..

[CIT0106] Vandiedonck C., Capdevielle C, Giraud M (2006). Association of the PTPN22*R620W polymorphism with autoimmune myasthenia gravis. Ann. Neurol..

[CIT0107] Chuang W. Y., Strobel P, Belharazem D (2009). The PTPN22gain-of-function+1858T(+) genotypes correlate with low IL-2 expression in thymomas and predispose to myasthenia gravis. Genes Immun..

[CIT0108] Kaya G. A., Coskun A. N, Yilmaz V (2014). The association of PTPN22 R620W polymorphism is stronger with late-onset AChR-myasthenia gravis in Turkey. PLoS One.

[CIT0109] Giraud M., Taubert R, Vandiedonck C (2007). An IRF8-binding promoter variant and AIRE control CHRNA1 promiscuous expression in thymus. Nature.

[CIT0110] Strobel P., Chuang W. Y, Chuvpilo S (2008). Common cellular and diverse genetic basis of thymoma-associated myasthenia gravis: role of MHC class II and AIRE genes and genetic polymorphisms. Ann. N. Y. Acad. Sci..

[CIT0111] Wang X. B., Pirskanen R, Giscombe R, Lefvert A. K (2008). Two SNPs in the promoter region of the CTLA-4 gene affect binding of transcription factors and are associated with human myasthenia gravis. J. Intern. Med..

[CIT0112] Gu M., Kakoulidou M, Giscombe R (2008). Identification of CTLA-4 isoforms produced by alternative splicing and their association with myasthenia gravis. Clin. Immunol..

[CIT0113] Smyth D. J., Plagnol V, Walker N. M (2008). Shared and distinct genetic variants in type 1 diabetes and celiac disease. N. Engl. J. Med..

[CIT0114] Kurko J., Besenyei T, Laki J (2013). Genetics of rheumatoid arthritis – a comprehensive review. Clin. Rev. Allergy Immunol..

[CIT0115] Ploski R., Szymanski K, Bednarczuk T (2011). The genetic basis of graves' disease. Curr. Genomics.

